# Bacteriological Spectrum and Drug Resistance Among Patients Associated With Bloodstream Infection in Intensive Care Units in the Affiliated Hospital of Jiaxing University From 2021 to 2023

**DOI:** 10.1155/cjid/7841940

**Published:** 2025-06-12

**Authors:** Yucheng Xie, Xiaochun Tan, Wei Wang, Bailong Hou, Minjie Mao, Xiaoqin Niu, Qinlong Yu, Weifeng Shen

**Affiliations:** ^1^Department of Clinical Laboratory, The First Hospital of Jiaxing, The Affiliated Hospital of Jiaxing University, Jiaxing, Zhejiang, China; ^2^Jiaxing Key Laboratory of Clinical Laboratory Diagnostics and Translational Research, The First Hospital of Jiaxing, The Affiliated Hospital of Jiaxing University, Jiaxing, Zhejiang, China

**Keywords:** antimicrobial resistance, bloodstream infection, intensive care unit, multidrug-resistant organisms, risk factors

## Abstract

Bloodstream infections (BSI) in ICU settings are associated with high morbidity, mortality, and healthcare costs. The ICU environment, with its high use of invasive devices and immunocompromised patients, fosters an increased risk for multidrug resistance (MDR) pathogens, complicating treatment strategies. Understanding the epidemiology and resistance patterns in these settings is essential for improving patient outcomes and guiding appropriate antimicrobial stewardship practices. This study retrospectively analyzed data from 640 blood culture samples collected in the ICU of the Affiliated Hospital of Jiaxing University between January 2021 and December 2023. The blood samples were appropriately collected and cultured. Matrix-assisted laser desorption/ionization time-of-flight (MALDI-TOF) mass spectrometry was employed to identify the isolated strains. Antimicrobial sensitivity was assessed using the VITEK2 system, the Epsilometer test (E-test), and the Kirby–Bauer disk diffusion method. All statistical analyses were conducted using IBM SPSS Statistics 22.0. A total of 391 bacterial pathogens (61.1%) were isolated. The predominant pathogens causing BSI were Gram-negative bacteria. The most prevalent pathogens during the period were coagulase-negative *Staphylococci* (CoNS, 17.1%), followed by *Klebsiella pneumoniae* (*K. pneumoniae*, 13.6%), *Enterococcus* spp (13.6%), *Escherichia coli* (*E. coli*, *1*2.3%), *Acinetobacter baumannii* (*A. baumannii*, 8.4%), and *Staphylococcus aureus* (*S. aureus*, 5.1%). Among the antibiotics tested, tigecycline, linezolid, vancomycin, and quinupristin/dalfopristin were effective against *Staphylococci* and *Enterococci*, although some CoNS strains exhibited resistance to vancomycin. Tigecycline showed effectiveness against the main gram-negative bacteria. Furthermore, multiple hospitalizations, comorbidity with diabetes, and the use of a central venous catheter were identified as significant risk factors for multidrug-resistant organisms (MDROs) in BSI cases. Pathogens isolated from the bloodstream of ICU patients exhibited significant drug resistance. We recommend strategies to mitigate the incidence of MDROs in BSI, including limiting the duration of hospital stays, closely monitoring underlying patient conditions, improving discharge plans, and strengthening transitional care, and prevent infections associated with central venous catheters.

## 1. Introduction

Antimicrobial resistance has emerged as one of the most pressing global public health challenges of our era [1]. Comprehensive estimates reveal that in 2019 alone, bacterial antimicrobial resistance contributed to 4.95 million mortality cases worldwide, with 1.27 million deaths directly attributable to antibiotic-resistant infections. Current projections paint an alarming picture: without immediate and coordinated global action, antimicrobial resistance-related fatalities could escalate to 10 million annually by 2050. This impending crisis not only threatens human health but also carries significant economic implications, potentially triggering an annual GDP reduction of 1.1%–3.8% across global economies [2, 3].

In China, bloodstream infections (BSI) have become a serious challenge due to the increasing problem of population aging [4]. MDRO infection is a common type of hospital infection, which is defined as bacterial strain resistance to three or more classes of antibiotics. According to the 2023 annual report from the China antimicrobial surveillance network (CHINET), the antimicrobial resistance profile of MDROs in China remains stable yet concerning. While surveillance data show a declining trend in methicillin-resistant *Staphylococcus aureus* (MRSA) isolation rates, concerning resistance patterns persist among Gram-negative pathogens. This epidemiological pattern presents significant challenges for clinical antimicrobial chemotherapy in China. The report emphasizes the critical need to enhance CHINET's monitoring infrastructure and implement coordinated strategies to curb the progression of antimicrobial resistance [5]. Various studies have explored the distribution and antibiotic resistance patterns of pathogens in BSI, revealing considerable differences in antibiotic use trends among provinces in China, which requires further research on regional data to support provincial antibiotic management [6–8].

Within the ICU settings, patients typically exhibit compromised immune function and routinely undergo treatment involving invasive devices. The prevalence of hospital-acquired infections can be 5–10 times higher exacerbated by the severe illnesses and the imprudent use of broad-spectrum antibiotics [9]. MDRO infections not only prolong hospital stays but also significantly increase the financial burden on patients [10]. The most concerning MDROs in the ICU are Gram-negative bacteria such as carbapenem-resistant *Acinetobacter baumannii* (CRAB), carbapenem-resistant Enterobacteriaceae, and carbapenem-resistant *Pseudomonas aeruginosa* and Gram-positive bacteria such as MRSA and vancomycin-resistant *Enterococcus*, which lead to drug resistance by producing resistant enzymes or carrying resistance genes. Therefore, it is imperative to implement robust infection prevention and control measures to curb the spread of MDROs in ICU settings.

The antimicrobial resistance patterns reported in other studies are not appropriate. It is necessary to establish the antimicrobial surveillance network of the Jiaxing model. This study aims to systematically analyze the prevalence, resistance profiles, and risk factors associated with MDROs in BSI cases, specifically within the context of the Affiliated Hospital of Jiaxing University. The objectives of this research are multifaceted: (1) to establish a detailed epidemiological profile of BSI pathogens, with an emphasis on MDROs, (2) to evaluate the evolving patterns of antibiotic resistance in various patient demographics, particularly in critically ill ICU patients, and (3) to identify key risk factors that contribute to the development and spread of MDROs in the hospital environment. This research uniquely contributes to the understanding of BSI in a regional hospital setting, offering insights into the local distribution of pathogens and the specific challenges posed by antimicrobial resistance. Additionally, this study will provide valuable information for tailoring empirical antibiotic therapy in the ICU, an area where timely and accurate treatment decisions are crucial for patient survival. Furthermore, the findings will support the development of targeted prevention and control policies, aiming to curb the rise of drug-resistant infections and improve clinical outcomes. This comprehensive approach, combining epidemiological data with actionable clinical insights, will be instrumental in refining infection control strategies at the Affiliated Hospital of Jiaxing University and potentially serve as a model for similar institutions in the region.

## 2. Methods

The emergency department of the Affiliated Hospital of Jiaxing University stands as the most technologically advanced and comprehensively equipped emergency care center in the region. Its clinical area spans nearly 5000 square meters, housing 76 multifunctional care units. The ICU specializes in the management of critically ill patients, including those with severe pneumonia, septic shock, multiorgan dysfunction syndrome, acute necrotizing pancreatitis, postcardiac surgical monitoring, and acute renal failure. The department is furnished with cutting-edge medical technologies and devices, enabling a significant enhancement in survival outcomes for critically ill patients through comprehensive multidisciplinary interventions.

### 2.1. Study Design

We analyzed blood culture samples from ICU patients with suspected BSI at the Affiliated Hospital of Jiaxing University (January 2021–December 2023). Patients of all ages and sexes with clinically suspected BSI were investigated. Only the initial strain isolated from each patient (first isolate per patient) was included to avoid bias from repeated testing or potential drug resistance development. Single-bottle positive samples and common contaminant such as *Micrococcus*, *Corynebacterium*, *Bacillus*, *Aerococcus*, and *Propionibacterium* were excluded as contaminants.

### 2.2. Blood Culture Collection Protocol

Blood samples were collected using Bact/Alert 3D system-compatible aerobic and anaerobic bottles adhering to the following protocols: Adults: Each bottle was filled with 8–10 mL of blood. A total of four to six bottles (aerobic and anaerobic combinations) were collected per patient for each infection event, with a total blood volume of ≥ 40 mL. Children: Blood volume was adjusted according to body weight, with a minimum of 1 mL per bottle (aerobic bottles were prioritized).

### 2.3. Incubation

Bottles were incubated at 35°C–37°C for 5 days. Blood culture systems (such as BACTECFX and VIRTUO) have built-in blood volume monitoring to observe the actual microbial metabolic activity through CO_2_ release or weight testing. Bottles with no microbial growth after 5 days were discarded without subculture.

### 2.4. Quality Control

Aseptic techniques were strictly followed to maintain a contamination rate of < 3% (ideally < 1%). A shunt is used to discard the first 1 mL of blood to reduce the risk of skin bacterial contamination. Multiple punctures were avoided, and contamination and false positive rates were reduced.

### 2.5. Sample Processing and Bacterial Identification

Upon detection of microbial growth, Gram staining and subculture onto selective media: Gram-positive cocci: Blood agar plates. Gram-negative bacilli: Blood agar, chocolate agar, and MacConkey agar. Fungi: Sabouraud dextrose agar and chromogenic *Candida* agar. Plates incubated aerobically at 35°C for 18–24 h. Single colonies are selected based on their shape and color for strain purification. Subculture was performed on fresh blood medium for isolation and antimicrobial susceptibility testing (AST).

### 2.6. Identification and AST

Bacterial identification and antimicrobial susceptibility test were performed following guidelines of the Clinical Laboratory Standards Association (CLSI). The antibiotic susceptibility data of pathogens were presented in percentage form. Microorganism identification was performed using matrix-assisted laser desorption/ionization time-of-flight mass spectrometry. AST was performed by the VITEK2 system, the E-test, and the Kirby–Bauer disk diffusion method. The following drugs were selected for the test: amikacin, ciprofloxacin, meropenem, piperacillin/tazobactam, gentamicin, cefepime, ceftazidime, tobramycin, imipenem, levofloxacin, compound sulfamethoxazole, tigecycline, ceftriaxone, colistin, ertapenem, aztreonam, amoxicillin/clavulanic acid, cefoxitin, rifampicin, oxacillin, erythromycin, clindamycin, quinoptin/dafoptin, Linezolid, moxifloxacin, penicillin G, tetracycline, vancomycin, ampicillin, high concentration of streptomycin, and high concentration of gentamicin. These data were interpreted according to the CLSI 2020 criteria (Clinical and Laboratory Standards Institute) [11]. Based on the CLSI criteria, drug susceptibility results can be reported in three categories: sensitive (S), intermediate (I), and resistant (R). Furthermore, the quality control strains including *Escherichia coli* ATCC25922, *S. aureus* ATCC25923*, Pseudomonas aeruginosa* ATCC27853, *Enterococcus faecalis* ATCC700327, and *Klebsiella pneumoniae* ATCC700324 were provided by the Shanghai Clinical Laboratory Center.

### 2.7. Statistical Analysis

Clinical data for all patients, encompassing age, sex, bed number, and results of bacterial susceptibility tests, were sourced from the laboratory information system and the Haiti electronic medical record system. The Shapiro–Wilk test assessed data normality. Continuous variables conforming to a normal distribution were expressed as mean ± standard deviation (SD) and analyzed between groups using independent sample *t*-tests. Continuous variables not adhered to a normal distribution, such as hospital stay duration and hemoglobin levels at admission, and data were presented as median (interquartile range, Q1–Q3) and analyzed using the nonparametric Mann–Whitney *U*-test. Categorical variables, including sex, age, hypertension status, hypoproteinemia, tumor, fracture, surgery, mixed infection, urinary tract infection, diabetes, central venous catheterization, and multiple hospitalization (≥ 2 unplanned hospital admissions within the 6 months preceding study enrollment, extracted from electronic health records and excluding routine outpatient visits), were reported as counts and compared using chi-square (*χ*^2^) tests, applying continuity corrections as required.

All statistical analyses were conducted using IBM SPSS Statistics 22.0 (IBM Corp., Chicago, Illinois, USA), with a significance level set at *p* < 0.05.

## 3. Result

### 3.1. Characteristics of Patients and Isolated Pathogen

A total of 640 patients participated in this study. The demographic characteristics of the participants are summarized in Table 1. Among these, 447 (69.8%) were male, and 193 (30.2%) were female. The majority of the patients were aged over 65 (61.7%), followed by those aged between 45 and 64 (29.0%), and the smallest group was those under 45 (9.3%). Out of the 640 blood samples collected, 391 (61.1%) yielded positive blood culture results. The sex distribution revealed that 129 (20.2%) of the 193 female patients had positive cultures. Similarly, 262 (40.9%) of the 447 male patients had positive cultures. Regarding age distribution, the highest infection rate was observed in patients older than 65 years (40.0%), followed by those aged 45–64 years (15.8%), and the lowest rate was among those younger than 45 years (5.3%).

**Figure 1:**
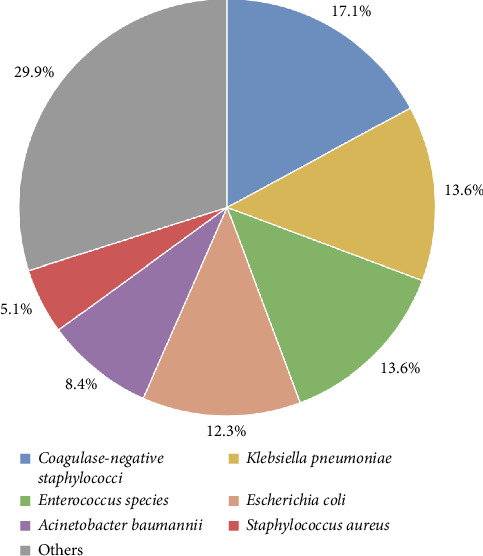
Distribution of main bacterial pathogens causing BSI in ICU patients at the Affiliated Hospital of Jiaxing University, 2021–2023. Note: The prevalence of bacterial pathogens isolated from 391 positive blood samples in ICU patients. Data are presented as percentages of total isolates.

Among the positive pathogens isolated in this study, 198 strains (50.7%) were Gram-negative bacteria, 153 strains (39.1%) were Gram-positive bacteria, and 40 strains (10.2%) were fungi.  Figure 1 illustrates that the predominant pathogens in blood samples during the whole period (2021–2023) was CoNS with 67 strains (17.1%), followed by *K. pneumoniae* with 53 strains (13.6%), *Enterococcus* spp. (including *Enterococcus faecium*, *Enterococcus faecalis*, and *Enterococcus gallinarum*) with 53 strains (13.6%), *E. coli with* 48 strains (12.3%), *A. baumannii with* 33 strains (8.4%), and *S. aureus with* 20 strains (5.1%).

**Figure 2:**
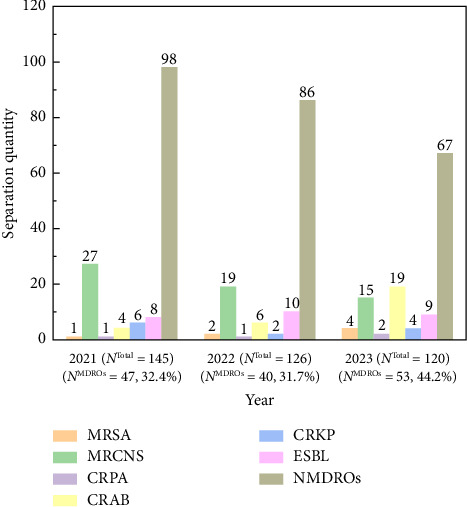
The distribution of MDROs and NMDROs causing BSI in patients from the ICU at the Affiliated Hospital of Jiaxing University, 2021–2023. Note: *N* is used to represent the number of positive pathogens each year. Abbreviations: MRSA, methicillin-resistant *Staphylococcus aureus*; MRCNS, methicillin-resistant coagulase-negative *Staphylococci*; CRPA, carbapenem-resistant *Pseudomonas aeruginosa*; CRAB, carbapenem-resistant *Acinetobacter baumannii*; CRKP, carbapenem-resistant *Klebsiella pneumoniae*; ESBL, extended-spectrum β-lactamase-producing *Enterobacterales*.

Figure 2 illustrates the distribution of MDROs from 2021 to 2023. In 2021, the highest annual isolation quantity of all pathogens was recorded at 145 strains, followed by 126 strains in 2022 and 120 strains in 2023. During the study period, a total of 140 strains of MDROs, constituting 35.8% of all isolates, were identified. Among these, 68 strains were Gram-positive bacteria, and 72 strains were Gram-negative bacteria. From 2021 to 2023, the number of MDROs rose by 12.8% (from 47 to 53), while the isolation rate increased by 11.8%, while the number of methicillin-resistant CoNS (MRCNS) strains decreased by about 50%, although their isolation rate remained unchanged. The isolation of CRAB strains in 2023 was nearly fourfold higher than in previous years. No significant changes were observed in the isolation rates of other Gram-negative bacteria.

### 3.2. Antimicrobial Resistance Pattern

The resistance pattern of major Gram-negative bacilli to antibiotics is presented in Table 1. Our study revealed that 26.4% of *K. pneumoniae* strains were resistant to cefepime, 32.1% to aztreonam, 30.8% to amoxicillin/clavulanic acid, approximately 25.0% to both imipenem and meropenem, and 11% to ertapenem. Regarding *E. coli*, more than 40.0% of the isolated strains demonstrated resistance to ceftriaxone and 44.9% to levofloxacin, and over 90.0% of isolated strains were sensitive to β-lactam antibiotics, with none exhibiting resistance to carbapenem. Isolated strains of *A. baumannii* exhibited strong resistance to all tested drugs, including third-generation cephalosporins (87.9% to ceftazidime and 84.8% to ceftriaxone), fourth-generation cephalosporins (81.8% to cefepime), carbapenem (84.8%–87.9%), piperacillin-tazobactam (82.1%), fluoroquinolones (approximately 82.0%), and aminoglycosides (32.1% to amikacin and 69.7% to gentamicin).

The resistance pattern of major Gram-positive bacilli to antibiotics is presented in Table 2. Regarding *S. aureus*, 95.0% were resistant to penicillin G, with a significant resistance rate to erythromycin (45.0%) and fluoroquinolones (30.0%–35.0%). Only 5.0% of the tested *S. aureus* strains showed resistance to clindamycin, and none were resistant to vancomycin. Among the CoNS strains, a minimal percentage (1.7%) showed resistant to vancomycin, while 91.7% were resistant to penicillin G, 88.3% to oxacillin, over 55% to fluoroquinolones, and 28.0% to clindamycin. No Gram-positive *Enterococcal* strains exhibited resistance to vancomycin, linezolid, tegacycline, and high concentration of streptomycin or gentamicin. The two antibiotics with the highest resistance rate in *Enterococcus faecalis* were erythromycin (60.0%) and penicillin G (31.6%). All strains of *Enterococcus faecium* were resistant to ampicillin and penicillin G, with 86.7% also resistant to erythromycin. The antimicrobial resistance rates for *Enterococcus faecalis* were lower than those for *Enterococcus faecium*, aligning with the findings reported by the CHINET in 2021 [12].

### 3.3. Factors Associated With MDROs in BSI

This study examined various clinical parameters and patient complications. We investigated candidate risk factors for the occurrence of MDROs in BSI, which included demographics (gender and age), medical history (previous admissions and hospitalization duration), clinical characteristics (hemoglobin levels at the time of infection and hypoalbuminemia), and comorbidities (diabetes, hypertension, malignancy, fractures, mixed infections, and urinary tract infections). Additionally, procedural factors such as surgeries and central venous catheterization were considered. We confirmed that the central venous catheter (*p*=0.002), multiple hospitalizations (*p*=0.003), urinary tract infection (*p*=0.013), and diabetes mellitus (*p*=0.026) were related to the occurrence of MDROs in BSI, as shown in Table 3. Nonsignificant factors are showed in Supporting 1.

### 3.4. Multifactorial Analysis of Risk Factors for the Occurrence of MDRO Infection

Multifactorial logistic regression analysis revealed that the central venous catheter, multiple hospitalizations, and diabetes mellitus were the independent risk factors for MDROs in BSI (Table 4). Notably, urinary tract infection did not increase the risk of MDRO colonization.

## 4. Discussion

BSI are among the most prevalent infections worldwide, resulting in significant mortality rates in hospitals. Given China's large patient population and increasing elderly demographic, effective measures to reduce infection can substantially alleviate both the physical and financial burdens on patients. The antimicrobial susceptibility of pathogens varies across different hospitals and regions. This study was conducted to identify the primary pathogens responsible for BSIs in ICU patients over a 3-year period, analyze their antibiotic resistance profiles, and identify risk factors associated with multidrug-resistant organisms.

Our investigation revealed that over 50% of ICU blood samples exhibited bacterial growth, aligning with the BSI rates reported by Sommerstein [13], yet it surpasses those noted in subsequent studies [14, 15]. Variations in the rates of positive blood cultures may stem from differences in result interpretation across hospitals, the volume of blood drawn (5 mL or 10 mL), and the number of samples collected (one or two culture bottles, i.e., aerobic/anaerobic pairs).

The primary pathogens responsible for BSI were identified as Gram-negative bacteria, contrasting previous findings that suggested a predominance of Gram-positive bacteria in BSI [16, 17]. However, numerous studies align with our results, indicating a significant presence of Gram-negative bacteria in the BSI case [18, 19]. We believe that the increased proportion of Gram-negative bacteria-related BSI could be attributed to enhanced preventative measures against central line-associated BSI, typically caused by Gram-positive bacilli, and the escalated use of drugs targeting antimicrobial resistances [20]. Variations in local infection prevention strategies and the prevalence of antimicrobial resistance may account for the differing trends observed across studies.

The strains most commonly associated with BSI were CoNS, corroborating findings from several studies [21, 22]. Over the past 2 decades, approximately 85% of CoNS isolates were initially regarded as pollutants during culture or collection processes. However, subsequent research has demonstrated their association with BSIs in patients with intravascular catheters and indwelling prosthetic device [23, 24]. Following CoNS, *K. pneumoniae* and *E. coli* were also frequently identified, aligning with other research that identified these pathogens, along with *S. aureus*, as primary causative agents of BSI [15]. Furthermore, studies focusing on MDROs causing infections at sites other than the bloodstream have found *K. pneumoniae* to be the predominant pathogen [25]. Additional research has highlighted *E. coli*, *S. aureus*, and *A. baumannii* as significant BSI pathogens [26–28]. Despite variations in regional pathogen prevalence, the primary causative agents of BSI remain consistent.

Antibiotics are widely recognized as the most effective means to combat infections. However, their empirical, indiscriminate, prolonged, or incorrect usage significantly impacts the prevalence of MDROs [29, 30]. In the ICU, the predominant Gram-negative bacilli demonstrated substantial resistance to most tested drugs, except tigecycline, and particularly to cephalosporins, aligning with findings reported in other countries [16, 17, 31, 32]. The misuse of antimicrobial agents such as cephalosporins and penicillin G has been shown to promote the emergence of ESBL-producing bacteria. These pathogens typically exhibit resistance not only to β-lactamase inhibitors but also to quinolones, aminoglycosides, and sulfonamides [33]. *E. coli* and *K. pneumoniae* were the most prevalent ESBLs-producing Enterobacteriaceae. These enzymes deactivate drugs containing β-lactam functional groups, including cephalosporins. In this study, 41.6% *E. coli* and 13.2% *K. pneumoniae* samples were identified as ESBLs-producing bacteria. β-lactamase inhibitors have been considered effective in managing ESBL-producing bacteria. Resistance rates of *K. pneumoniae* to piperacillin/tazobactam and amoxicillin-clavulanic acid were found to be 28.3% and 30.8%, respectively. This high resistance rate may be attributed to the recent increase in β-lactamase activity [34].

The widespread dissemination of carbapenem-resistant bacteria presents a significant concern, as carbapenems are the primary antibiotics used to treat these infections in the ICU [35, 36]. In our study, both *K. pneumoniae* and *A. baumannii* exhibited high levels of carbapenem resistance. Notably, infections caused by *A. baumannii* in ICUs have quadrupled, making it the most prevalent infection in 2023. The positive rate for CRAB reached 87.9%. The significant increase in CRAB cases may be related to the relaxation of China's epidemic prevention policies at the end of 2022, leading to a rise in COVID-19 infections. Several studies have reported a notable rise in CRAB infections during the pandemic in ICUs globally. For instance, a study from Italy highlighted an increased incidence of CRAB infections among COVID-19 patients, attributed to factors such as immunosuppression, the extensive use of broad-spectrum antibiotics, and challenges in implementing infection control measures [37]. Furthermore, another study suggested that the heightened demand for intensive care and mechanical ventilation among COVID-19 patients contributed to a higher risk of infections caused by multidrug-resistant organisms, including CRAB [38]. Although these studies were primarily conducted in international settings, their findings are relevant to Chinese healthcare facilities. During the COVID-19 pandemic, the surge in ICU admissions and the strain on healthcare resources may have compromised the execution of infection prevention and control measures, thereby facilitating the spread of CRAB infection [39].

The high resistance to penicillin G observed in Gram-positive bacteria aligns with findings from studies in Jimma [40] and Afghanistan [41]. Similarly, our results showed that nearly all Gram-positive bacteria were susceptible to vancomycin, consistent with other research [42]. However, a small incidence of vancomycin-resistant CoNS was detected in this study.

Multiple antimicrobial stewardship initiatives have been implemented in Zhejiang Province in 2021, requiring the standardization of antibacterial drug use in medical institutions. The policy emphasized strict control over the utilization intensity of high-risk antibiotics, including carbapenems, tigecycline, and polymyxins, while enforcing specialized registry management for agents such as polymyxin B and colistin. Despite these restrictions, the isolation rate of MDROs shows growth trend, and CRAB in the ICU in our study was nearly four times higher in 2023, highlighting challenges in policy implementation. Clinicians may still rely heavily on carbapenems for critically ill patients, limiting the policy's effectiveness in reducing resistance. Data on actual carbapenem use in the ICU was not available in this study, according to the study of Lamoth; the use of carbapenems was particularly high in the ICU, significantly exceeding the recommended daily dose [43]. Our research indicates that Gram-negative bacteria exhibit significant sensitivity to tigecycline. Restrictions on tigecycline use have effectively slowed the progression of bacterial resistance. However, the study also noted that tigecycline use might be associated with increased mortality in critically ill patients, highlighting the need to balance efficacy and safety in policy implementation [44]. Compared to other regions, Zhejiang's antimicrobial management policies demonstrate notable rigor in design and execution. Nevertheless, compared to regions such as Shanghai and Beijing, which have implemented real-time surveillance systems, Zhejiang lags in data feedback and dynamic adjustment mechanisms, making it harder to respond promptly to emerging resistance trends.

Several risk factors contribute to the prevalence of MDROs. Our research identified multiple hospitalizations, diabetes, and central venous catheterization as independent risk factors for MDROs in BSI. Glycosuria may facilitate bacterial growth, while hyperglycemia can impair neutrophil and macrophage function. Additionally, weakened immune responses and end-organ diseases that impair tissue oxygenation may exacerbate hyperglycemic disturbances [45, 46]. Catheter-associated Gram-negative bacteremia typically necessitates the removal of the catheter to prevent recurrent infections. Studies demonstrates that the rate of central line-associated BSI decreased from 2.8 to 0.9 per 1000 central venous catheter days following the removal of femoral arterial and hemodialysis central venous catheters. Subcutaneous catheters are associated with the lowest risk of infection [47]. Multiple hospitalizations and prolonged treatment durations are associated with increased risk of MDRO infections. An analysis of patients with bacteremia due to Enterobacteriaceae infections indicates that shorter treatment durations (6–10 days) are associated with a reduced risk of subsequent MDRO infections [48]. While urinary tract infections can lead to secondary BSIs via bacterial translocation, particularly in patients with indwelling catheters or urinary obstruction [46], our findings contrast with the existing literature. Catheter-associated urinary tract infections are well-documented to disrupt the urinary tract's natural defense mechanisms, increasing the risk of bacteremia and MDRO acquisition. However, in our cohort, indwelling catheter use was not independently associated with MDROs-BSI, contrary to prior reports. This discrepancy may stem from differences in patient populations, catheter care protocols, or local antimicrobial stewardship practices. For instance, stringent aseptic techniques and early antibiotic treatment after catheter removal in our setting might have mitigated the risk of MDRO dissemination. The discovery of this phenomenon provides new insights into understanding the epidemiological characteristics of infections and drug resistance mechanisms within local ICUs, while also facilitating the formulation of more targeted infection prevention/control strategies and antimicrobial drug utilization protocols in clinical practice. Of course, this represents only a preliminary interpretation and exploration, and we plan to conduct more in-depth studies to validate and refine these conclusions.

These findings underscore the imperative for precision infection prevention strategies in critical care settings. We propose a multidimensional intervention framework targeting risk factors. Effective prevention of central venous catheter-associated BSI relies primarily on two strategies: standardizing catheter insertion practices and incorporating advanced insertion technologies [49, 50]. Adherence to stringent hand hygiene and aseptic techniques during the catheter insertion phase is crucial. The use of antimicrobial-coated catheters, such as those impregnated with chlorhexidine, can help prevent microbial colonization. Additionally, the implementation of ultrasound-guided central venous catheterization can significantly reduce the incidence of infectious complications by improving the accuracy of vascular access and minimizing procedural trauma. For patients requiring prolonged catheterization, meticulous attention to dressing management, catheter securement, hub disinfection, and the use of antimicrobial lock solutions is essential to mitigate infection risk. Generally, unnecessary catheters should be removed promptly to minimize the duration of catheter placement. Preventing each CABSI can save nearly $10,000. Hyperglycemia may promote bacterial growth and reduce the efficacy of antibiotics. Proper control of blood glucose levels in ICU patients, particularly maintaining nondiabetic patients' blood glucose concentrations between 120 and 140 mg/dL, can reduce infection-related complications [51]. We recommend enhancing blood glucose monitoring, optimizing insulin use, and minimizing glucose fluctuations in the ICU to decrease the incidence of ICU-related BSI. Prolonging the length of stay of ICU patients or repeated hospital admissions will increase the patients' risk of exposure to MDROs. By improving discharge plans, strengthening transitional care (such as post-ICU clinics), conducting telemedicine follow—ups after patients are discharged, and implementing rehabilitation programs, the readmission rate can be effectively reduced, thus indirectly reducing the cumulative infection risk caused by repeated hospitalizations [52].

This research has several limitations. Firstly, it is a single-center study conducted at the largest general hospital in Jiaxing. Consequently, the results may not be representative due to varying sensitivity rates across different hospitals and units. Secondly, the clinical data available for this study are limited, lacking information on recent hospitalizations, clinical treatment records, and past antibiotic usage. Furthermore, this study only analyzed the sensitivity and drug resistance patterns of the six most prevalent pathogens in BSI; other microorganisms were not considered. Previous studies have indicated that comprehensive clinical conditions and historical data on antibiotic use could facilitate the identification of risk factors for antimicrobial resistance and support the development of highly reliable treatment recommendations. Therefore, a multicenter prospective study is necessary to address the current gaps in evidence related to BSI.

## 5. Conclusion

The resistance of pathogens isolated from blood samples of ICU patients is increasingly concerning. Our findings will aid clinicians in the ICU to better understand the resistance status and risk factors associated with BSI. MDROs limit treatment options and contribute to increased mortality rates. It is crucial for medical laboratories to continuously monitor the resistance trends of pathogens. Infection prevention measures should be strengthened in clinical units, including the monitoring of antibiotic use to prevent the spread of MDROs.

## Figures and Tables

**Table 1:** The characteristic classification and positive rate of patients from ICU at the Affiliated Hospital of Jiaxing University, 2021–2023.

Category	Number of samples collected (*N*, %)	Number of positive samples (*N*, %)
Sex		
Male	447 (69.8)	262 (40.9)
Female	193 (30.2)	129 (20.2)
Total	640 (100.0)	391 (61.1)
Age		
≤ 44	60 (9.3)	34 (5.3)
45–64	185 (29.0)	101 (15.8)
≥ 65	395 (61.7)	256 (40.0)
Total	640 (100.0)	391 (61.1)

**Table 2:** Antibiotic resistance rates of major Gram-positive bacteria from the ICU at the Affiliated Hospital of Jiaxing University, 2021–2023.

Antibiotic	Classes	*S. aureus* (%)	CoNS (%)	*E. faecium* (%)	*E. faecalis* (%)
Ampicillin	β-lactams	—^a^	—^a^	**100.0**	0.0
Oxacillin	β-lactams	35.0	**88.3**	—^a^	—^a^
Penicillin G	β-lactams	**95.0**	**91.7**	**100.0**	31.6
Gentamicin	Aminoglycosides	0.0	16.9	—^a^	—^a^
High concentration of streptomycin	Aminoglycosides	—^a^	—^a^	0.0	0.0
High concentration of gentamicin	Aminoglycosides	—^a^	—^a^	0.0	0.0
Ciprofloxacin	Fluoroquinolones	35.0	**70.0**	—^a^	—^a^
Levofloxacin	Fluoroquinolones	35.0	**71.7**	—^a^	—^a^
Moxifloxacin	Fluoroquinolones	30.0	**56.7**	—^a^	—^a^
Erythromycin	Macrolides	45.0	**73.3**	**86.7**	**60.0**
Clindamycin	Lincosamides	5.0	28.0	—^a^	—^a^
Tigecycline	Glycylcyclines	0.0	0.0	0.0	0
Vancomycin	Glycopeptides	0.0	1.7	0.0	0.0
Linezolid	Oxazolidinones	0.0	—^a^	0.0	0.0
Rifampicin	Ansamycins	0.0	8.3	—^a^	—^a^
Tetracycline	Tetracyclines	5.0	16.7	—^a^	—^a^
Compound sulfamethoxazole	Sulfonamides	5.0	35.0	—^a^	—^a^
*Quinupristin*/dalfopristin	Streptogramins	0.0	—^a^	0.0	0.0

*Note:* Data are presented as percentages of resistant isolates. Bold values indicate resistance rates ≥ 50%.

Abbreviations*:* CoNS, *Coagulase-negative Staphylococci*; *E. faecalis*, *Enterococcus faecalis*; *E. faecium*, *Enterococcus faecium*; *S. aureus*, *Staphylococcus aureus*.

^a^Not applicable (no data available or not tested).

**Table 3:** Analysis of influencing factors of MDROs of BSI.

Characteristics	NMDRO (*N* = 250) (*N* = 250)	MDRO (*N* = 141) (*N* = 141)	*p* value
*Central venous catheterization*			
Yes	105	82	0.002
No	145	59	

*Multiple hospitalization*			
Yes	59	53	0.003
No	191	88	

*Urinary tract infection*			
Yes	42	11	0.013
No	208	130	

*Complicated with diabetes*			
Yes	75	58	0.026
No	175	83	

*Note:* Nonsignificant factors are showed in Supporting file 1. MDRO, multidrug-resistant organism; NMDRO, nonmultidrug-resistant organism.

**Table 4:** Multivariate logistic regression analysis for the infection of MDRO of BSI.

Parameters	β	SE	Wald	OR	95% CI	*p* value
Constant term	−1.196	0.201	35.265	0.302	NA	< 0.001
Central venous catheterization	0.642	0.222	8.353	1.900	1.232–2.947	0.004
Complicated with diabetes	0.585	0.234	6.253	1.794	1.135–2.843	0.012
Urinary tract infection	−0.949	0.375	6.406	0.387	0.178–0.782	0.011
Multiple hospitalization	0.685	0.238	8.269	1.983	1.244–3.168	0.004

*Note:* MDRO, multidrug-resistant organism; Wald, Wald statistic; β, regression coefficient.

Abbreviations: N/A, not applicable; OR, odds ratio; SE, standard error; 95% CI, 95% confidence interval.

## Data Availability

The data used to support the findings of this study are available from the corresponding author upon reasonable request.

## Supporting Information

In this study, we analyzed the risk factors associated with MDRO infections. To concisely present the key findings, only factors demonstrating statistically significant correlations are listed in Table 3, while other analyzed variables are provided in the Supporting Information.
