# Supplementary material for: Bacteriological Spectrum and Drug Resistance Among Patients Associated With Bloodstream Infection in Intensive Care Units in the Affiliated Hospital of Jiaxing University From 2021 to 2023
Source: Can J Infect Dis Med Microbiol. 2025 Jun 12;2025:7841940. doi: 10.1155/cjid/7841940 (PMC12178772; doi:10.1155/cjid/7841940)
Supplement: Supporting Information — Additional supporting information can be found online in the Supporting Information section. [file 7841940.f1.docx]

| **Characteristics** | **NMDRO (N=250) (N=250)** | **MDRO (N=141)**  **(N=141)** | **P-value** |
| --- | --- | --- | --- |
| Sex |  |  |  |
| Male | 165 | 98 | 0.478 |
| Female | 85 | 43 |  |
| Age |  |  |  |
| >60 | 175 | 100 | 0.848 |
| ≤60 | 75 | 41 |  |
| Hospital stays (days) [d, M (Q1, Q3)] | 4.0 (1.0, 16.0) | 5.0 (1.0, 13.0) | 0.779 |
| Hemoglobin at infection (g/L) [d, M (Q1, Q3)] | 92.0 (78.0, 106.75) | 96.0 (81.75, 116.0) | 0.074 |
| Hypoproteinemia |  |  |  |
| Yes | 102 | 51 | 0.368 |
| No | 148 | 90 |  |
| Hypertension |  |  |  |
| Yes | 115 | 66 | 0.878 |
| No | 135 | 75 |  |
| Malignant tumor |  |  |  |
| Yes | 40 | 17 | 0.289 |
| No | 210 | 124 |  |
| Fracture |  |  |  |
| Yes | 45 | 28 | 0.651 |
| No | 205 | 113 |  |
| Surgery |  |  |  |
| Yes | 108 | 61 | 0.991 |
| No | 142 | 80 |  |
| Mixed infection |  |  |  |
| Yes | 70 | 35 | 0.496 |
| No | 180 | 106 |  |

**Supplement 1.** Analysis of Influencing factors of MDRO of BSI

**Note**: Categorical variables are expressed as frequencies; acontinuous variables (e.g., hemoglobin) are reported as median (interquartile range, Q1–Q3).

**Abbreviations**: MDRO, multidrug-resistant organism; NMDRO, non-multidrug-resistant organism.
